# Evaluation of Long-Term Key Outcomes and Safety in Pulmonary Embolism: The EKOS-PE Study

**DOI:** 10.1016/j.jscai.2025.103712

**Published:** 2025-07-23

**Authors:** Sameh Sayfo, Taylor Pickering, Ghadi Moubarak, Kyle M. McCullough, Cody W. Dorton, Mohamad Bader AboHajar, Tanushree Prasad, Colleen Parro, Maya Raghunathan, Pratham Bhavikati, Swapnil Gupta, Madhura Kapil Shah, J. Michael DiMaio, Sibi Thomas, Karim Al-Azizi, Dennis Gable, John Kedora, Chadi Dib, Srini Potluri, Molly Szerlip, Timothy J. George, Zachary P. Rosol, Subhash Banerjee

**Affiliations:** a Department of Cardiology, Baylor Scott & White The Heart Hospital − Plano, Plano, Texas; b Baylor Scott & White Research Institute, Baylor Scott & White The Heart Hospital − Plano, Plano, Texas; c Department of Cardiothoracic Surgery, Baylor Scott & White The Heart Hospital − Plano, Plano, Texas; d Department of Biomedical Engineering, Texas A&M University, College Station, Texas; e Department of Vascular Surgery, Baylor Scott & White The Heart Hospital − Plano, Plano, Texas; f Department of Cardiology, Baylor University Medical Center, Dallas, Texas

**Keywords:** catheter-directed thrombolysis, EkoSonic Endovascular System therapy, pulmonary embolism, quality of life outcomes, right ventricular dysfunction

## Abstract

**Background:**

Pulmonary embolism (PE) is a major cause of morbidity and mortality, particularly in massive and submassive cases that lead to right ventricular (RV) strain and long-term complications. The EkoSonic Endovascular System (EKOS) offers a catheter-directed thrombolytic treatment option for patients with contraindications to systemic thrombolysis, but data on long-term outcomes remain limited. We aimed to evaluate long-term mortality, RV function, and quality of life (QoL) in patients with massive or submassive PE who were treated with EKOS therapy.

**Methods:**

The EKOS-PE is a retrospective cohort study of 137 patients with massive or submassive PE who underwent EKOS therapy within a single health care system from 2020 to 2024. The primary outcome was all-cause mortality; secondary outcomes included changes in the RV-to-left ventricular (LV) (RV/LV) ratio, residual RV dysfunction, and QoL as assessed using the Pulmonary Embolism Quality of Life questionnaire.

**Results:**

All-cause mortality was 7.2% at a mean follow-up of 26.5 ± 17.2 months. During the index hospitalization, 1 retroperitoneal bleed (0.7%) and 1 ischemic stroke (0.7%) were observed. The mean RV/LV ratio decreased from 1.13 ± 0.24 to 0.83 ± 0.19 (*P* < .01). No residual RV dysfunction was evident in 105 (75.5%) patients, whereas 16 (11.5%) exhibited moderate to severe residual dysfunction. Of 52 respondents who completed the QoL survey at a mean follow-up of 37.2 ± 12.1 months, minimal residual symptoms, limited functional interference, and improved perceived lung health were reported.

**Conclusions:**

The EKOS therapy is associated with significant long-term improvement in RV function, low mortality, and favorable perceived QoL, supporting its use in massive and submassive PE, aligning with current guideline recommendations for high-risk patients.

## Introduction

It is estimated that acute pulmonary embolism (PE) is responsible for more than 100,000 deaths among hospitalized patients in the United States each year.^1^ PE is classified as high-risk (massive), intermediate-risk (submassive), or low-risk based on clinical presentation, right ventricular (RV) strain, and hemodynamic stability.^2^ Massive PE is characterized by sustained hypotension, shock, or cardiac arrest, whereas submassive PE presents with RV dysfunction and/or myocardial injury without overt hemodynamic compromise. Patients with low-risk PE demonstrate neither hemodynamic instability nor RV dysfunction. Historically, the mortality risk with submassive PE ranged from 5% to 25%, whereas massive PE carried an 18% to 65% rate of mortality.^3^ Additionally, survivors are prone to long-term complications, including recurrent venous thromboembolism (25%-33%), postthrombotic syndrome (50%), and chronic thromboembolic pulmonary hypertension (0.5%-4%), which collectively impair quality of life (QoL).^4^

Treatment of acute PE has evolved significantly since the landmark clinical trial by Barritt and Jordan, published in the *Lancet* in 1960, which concluded that heparin reduced the risk of death following PE.^5^ The overall mortality rate for all hospitalized patients decreased from 8.3% in 1999 to 4.4% in 2010, mainly due to direct oral anticoagulant therapy.^6^ Current guidelines from the European Society of Cardiology and the American Heart Association recommend anticoagulation and systemic thrombolysis as first-line treatments for high-risk patients, a class 1 indication.^7^, ^8^, ^9^, ^10^ However, systemic thrombolysis carries a risk of major bleeding, necessitating alternative strategies for patients with contraindications.^9^ Catheter-directed thrombolysis (CDT), such as the EkoSonic Endovascular System (EKOS; Boston Scientific), provides targeted thrombolytic therapy combined with ultrasound-assisted fibrinolysis, offering a promising option to mitigate the risks associated with systemic thrombolysis.^7^^,^^8^^,^^11^

Following the Food and Drug Administration approval in 2014, several pivotal trials, including ULTIMA, SEATTLE II, and OPTALYSE PE, have established the short-term safety and efficacy of EKOS in reducing RV strain.^12^, ^13^, ^14^, ^15^ Similarly, a recent retrospective study demonstrated a reduction in RV strain and fewer complications in the 6 months following intervention with EKOS for patients with submassive PE.^16^ However, limited data exist on the durability and long-term outcomes of the EKOS therapy. To address this gap, the EKOS-PE study aimed to evaluate the long-term clinical outcomes, including RV function and QoL, in patients with massive and submassive PE treated with the EKOS therapy.

## Methods

The EKOS-PE study is a retrospective cohort study that included adult patients with contrast-enhanced CT-confirmed acute massive or submassive PE who underwent EKOS CDT within the Baylor Scott & White Healthcare system from March 2020 to March 2024. Massive PE was defined as a documented PE with subsequent syncope, systemic hypotension (systolic blood pressure <90 mm Hg), cardiogenic shock, or resuscitated cardiac arrest. Submassive PE was defined by the presence of RV dysfunction (RV/left ventricular [LV] ratio ≥1.0 on echocardiography, or troponin I >0.4 ng/mL) without hemodynamic shock. Patients with contraindications to systemic thrombolysis were prioritized for CDT. All patients received the EKOS therapy with dosing protocols adherent to institutional guidelines for PE based on the OPTALYSE dosing protocol, with adjustments based on clinical stability and bleeding risk for each patient.^14^ Patients were monitored during procedure and postprocedure for hemodynamic stability in an intensive care setting and were transitioned to therapeutic anticoagulation following successful thrombolysis.

Demographic characteristic, clinical, laboratory, and echocardiographic data were extracted from electronic medical records. Echocardiographic parameters collected focused on RV/LV diameter ratios and severity of RV dysfunction before and after the EKOS therapy. The primary outcome was all-cause mortality following EKOS intervention. Secondary outcomes included changes in RV/LV diameter ratios, residual RV dysfunction, all-cause 30-day readmission rate, 1-year PE-related readmission rates, and QoL as assessed by the validated Pulmonary Embolism Quality of Life (PEmb-QoL) questionnaire.^17^

Continuous variables were summarized as mean ± SD or median with IQR for nonnormally distributed data. Categorical variables were reported as frequencies and percentages. Changes in RV/LV diameter ratios were analyzed using paired *t* tests, with statistical significance set at *P* <.05. All analyses were performed using R version 4.4.1 (R Core Team).

### Ethical statement

Institutional review board (IRB) approval was obtained before initiating data collection (IRB No. 023-085). A waiver of written informed consent was granted for both the retrospective and prospective components due to the minimal risk posed by the study design. All patients who participated in the QoL questionnaire, however, were informed that completion of the survey was voluntary and that their responses would be used for research purposes. The telephone script used to administer the QoL questionnaire, detailing the information provided to participants, is included in the Supplemental Appendix 1.

## Results

We identified 137 patients with massive or submassive PE who underwent the EKOS therapy from March 2020 to March 2024. The mean age was 60.8 ± 14.5 years, with a slight female predominance (55.5%). Risk factors for PE were common: 26 patients (19.0%) had a history of cancer, 9 (6.6%) were immobile (eg, paraplegia or quadriplegia), 13 (9.5%) underwent a medical procedure within 30 days of diagnosis, and 26 (19.0%) had been hospitalized in the prior 30 days. Concomitant deep vein thrombosis was found at the time of PE diagnosis in 81 (59.1%) patients (Table 1). At presentation, 17 (12.4%) patients met the criteria for massive PE: 12 presented with syncope, 3 with persistent hypotension, 1 with cardiogenic shock, and 1 in cardiac arrest. The remaining 120 (87.6%) patients were classified as submassive. Of these, 43 had severe RV dysfunction (25 also had elevated troponin I), 26 had moderate RV dysfunction (16 with elevated troponin), 25 had mild RV dysfunction (16 with elevated troponin), and 26 had no RV dysfunction (12 with elevated troponin). The median pulmonary embolism severity index score was 79.0 (IQR, 65.0-98.5).^18^

**Table 1:** Baseline patient characteristics and comorbidities.

Characteristics	N = 137
Age, y	60.78 ± 14.46
Body mass index	37.38 ± 10.28
Female sex	76 (55.47)
Male sex	61 (44.53)
Hypertension	98 (71.5)
Hyperlipidemia	62 (45.3)
Diabetes	41 (29.9)
Coronary artery disease	19 (13.9)
Peripheral arterial disease	7 (5.1)
Congestive heart failure	15 (10.9)
Chronic obstructive pulmonary disease	10 (7.3)
Asthma	13 (9.5)
Tobacco use	24 (17.5)
COVID-19 positive	4 (2.9)
Immobility	9 (6.6)
Connective tissue disorder	0 (0)
History of malignancy	26 (19.0)
Exogenous hormone use	26 (19)
Non-PE procedure <30 days	13 (9.5)
Hospitalization <30 days	26 (19.0)
Massive pulmonary embolism on admission	34 (24.8)
PESI score	79 (65-98.5)

Categorical data are described as n (%). Continuous data are described as mean ± SD or median (IQR).

PESI, pulmonary embolism severity index.

At a mean follow-up of 26.5 ± 17.2 months, the all-cause mortality rate was 7.3% (10/137). Of these deaths, 1 was attributed to recurrent PE occurring more than 2 years postprocedure, 1 to respiratory failure secondary to interstitial lung disease, 1 to anoxic brain injury, and 2 to sepsis. The remaining deaths were of unknown etiology. Fifteen patients were lost to 30-day follow-up, and 31 were lost to 1-year follow-up. The 30-day all-cause readmission rate was 5.0%, with 1 readmission (0.7%) related to PE. During the index hospitalization, 1 patient experienced a retroperitoneal bleed (0.7%), and another suffered acute punctate watershed infarctions on postprocedure day 4 (0.7%), with no residual neurological deficits noted. No patients experienced a postprocedure myocardial infarction.

**Figure 1:**
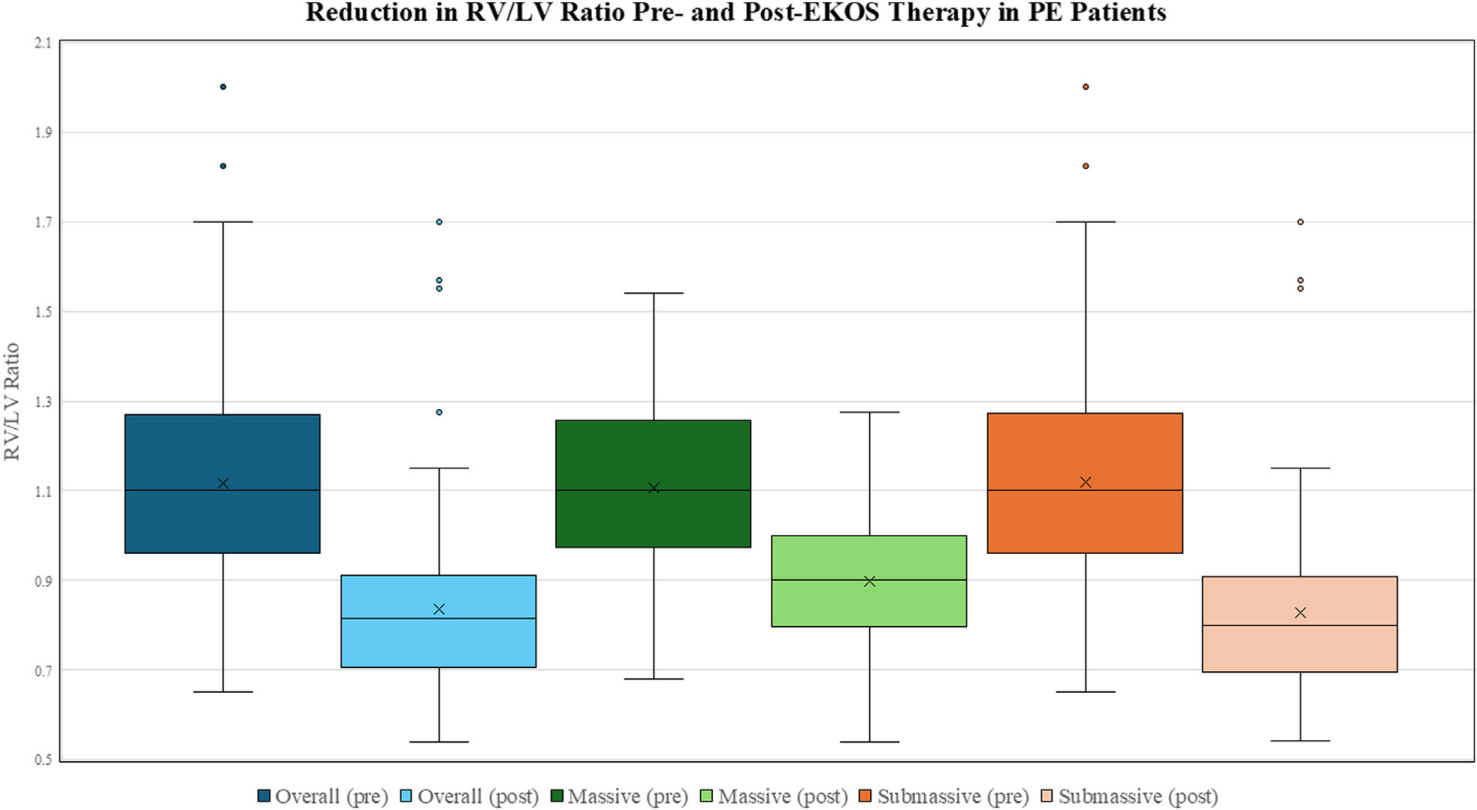
**Box-and-whisker plots illustrating the right ventricular (RV) to left ventricular (LV) ratio as measured by an echocardiogram before (pre) and after (post) the EkoSonic Endovascular System (EKOS) therapy.** Data are stratified by overall cohort, massive pulmonary embolism (PE), and submassive PE.

**Figure 2:**
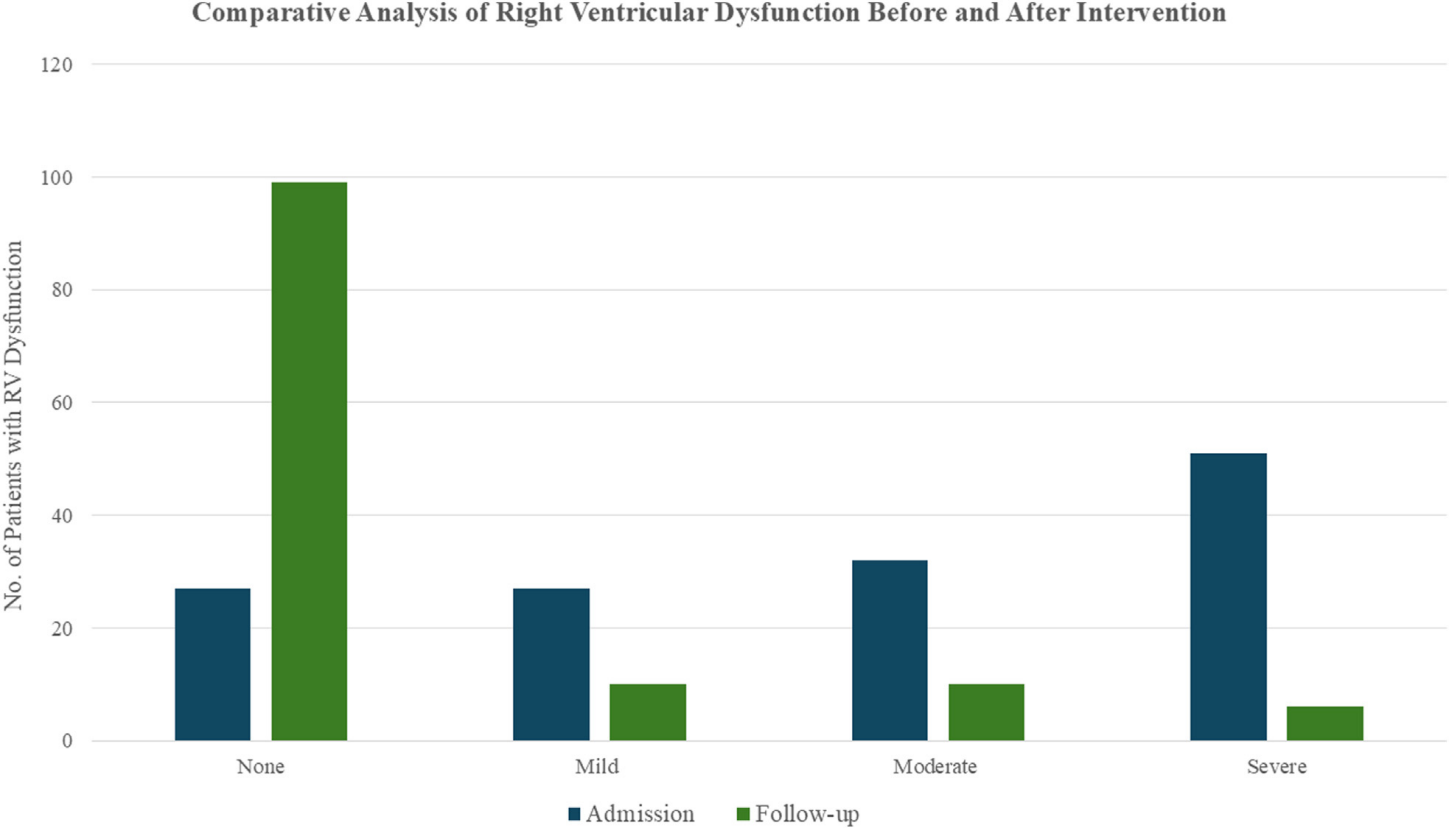
**Bar chart comparing the distribution of right ventricular(RV) dysfunction severity before and after the EkoSonic Endovascular System therapy.**

At presentation, the overall baseline mean RV/LV ratio was 1.12 ± 0.24. Stratified by PE severity, both massive and submassive PE groups had a similar mean baseline RV/LV ratios of 1.11 ± 0.23 and 1.12 ± 0.24, respectively. Of the 125 (91.2%) patients with postprocedure echocardiograms, the mean RV/LV ratio decreased to 0.84 ± 0.19, corresponding to a median reduction of 26.7% (12.5-37.0) (*P* < .01) at a mean follow-up of 5.22 ± 7.77 months (Figure 1). When examined separately, both submassive and massive groups demonstrated significant reductions from their respective baselines. Patients with massive PE had a postprocedure RV/LV ratio of 0.90 ± 0.17 (median reduction 21.3% [9.8%-29.0%], *P* < .01), whereas those with submassive PE had a postprocedure RV/LV ratio of 0.83 ± 0.19 (median reduction 27.2% [14.4%-37.5%], *P* < .01) (Table 2). Overall, RV dysfunction was observed in 80.3% of patients, with severe dysfunction in 37.2%, moderate dysfunction in 23.4%, and mild dysfunction in 19.7% prior to intervention. At follow-up, 79.2% of patients exhibited no evidence of RV dysfunction, whereas 8% had mild dysfunction, 8% had moderate dysfunction, and 4.8% remained with severe dysfunction (Figure 2).

**Table 2:** Admission and follow-up echocardiogram results.

Echocardiographic results	
Admission echocardiogram	N = 137
RV/LV ratio	
Overall	1.12 ± 0.24 1.1 (0.96-1.27)
Massive	1.11 ± 0.23 1.10 (1.00-1.24)
Submassive	1.12 ± 0.24 1.10 (0.96-1.27)
RV dysfunction	
None	27 (19.7)
Mild	27 (19.7)
Moderate	32 (23.4)
Severe	51 (37.2)
Follow-up echocardiogram	N = 125
RV/LV ratio	
Overall	0.84 ± 0.19 0.82 (0.71-0.91)
Massive	0.90 ± 0.17 0.90 (0.81-1.00)
Submassive	0.83 ± 0.19 0.80 (0.70-0.91)
Percent change of RV/LV ratio	
Overall	22.8 ± 22.5 26.7 (12.5-37.0)
Massive	17.6 ± 23.3 21.3 (9.8-29.0)
Submassive	23.5 ± 22.4 27.2 (14.4-37.5)
Right ventricular dysfunction	
None	99 (79.2)
Mild	10 (8.0)
Moderate	10 (8.0)
Severe	6 (4.8)

Categorical data are described as n (%). Continuous data are described as mean ± SD or median (IQR).

LV, left ventricular; RV, right ventricular.

Quality of life outcomes were assessed using the PEmb-QoL questionnaire in 52 patients at a mean follow-up of 37.2 ± 12.1 months (Supplemental Table 1). The remaining patients either declined participation in the survey or were unavailable despite repeated contact attempts. Overall, participants reported substantial improvements in respiratory symptoms and functional capacity following the procedure. Most patients indicated minimal or no ongoing pulmonary complaints. Median scores for pain behind or between the shoulder blades, back pain, sensation of pressure, a feeling that “there is still something there,” burning sensations, and nagging feelings in the lungs were all 5 (5-5), corresponding to “never” experiencing these symptoms. Mild chest pain (5 [4-5]) and breathlessness (5 [3-5]) were reported by some, but remained relatively infrequent or absent for most. Half of the respondents (50%) never experienced lung symptoms at any time of the day, whereas 23.1% noted that symptoms, if present, could arise at any time. When asked to compare their current lung status to their preprocedure condition, 61.5% felt their lungs were “much better” now, and another 7.7% felt they were “somewhat better.” In terms of activity limitations, the median score for routine home tasks, social activities, and moderate physical exertion (eg, walking a few hundred yards) was 3 (3-3), indicating “no limitation at all.” More strenuous activities, such as running or climbing multiple flights of stairs, had a slightly broader range (3 [1-3]), reflecting mild-to-moderate limitations in a subset of patients. Despite these improvements, some individuals still experienced work or daily activity restrictions related to pulmonary symptoms. Specifically, 19.2% cut down on the amount of time spent on work/activities, 25% accomplished less than desired, and 26.9% felt limited in the kind of tasks they could perform. Nevertheless, 65.4% of patients reported no interference in their normal social activities, and 69.2% had no pain around the shoulder blades or chest “at present.” Over half (53.8%) indicated no breathlessness, though a small proportion reported more severe shortness of breath. Finally, in the 4 weeks preceding the survey, patients remained somewhat concerned about the possibility of recurrent PE (4 [3-6]) and about potentially stopping anticoagulation therapy (4.5 [2-6]).

## Discussion

The EKOS-PE study found that the EKOS therapy for massive and submassive PE significantly reduced the RV/LV diameter ratio, improved long-term RV function, and was associated with a low all-cause mortality rate and minimal residual symptoms. These findings expand on current evidence supporting EKOS as an effective and safe intervention for patients at high-risk or with contraindications to systemic thrombolysis (Central Illustration).

**Central Illustration:**
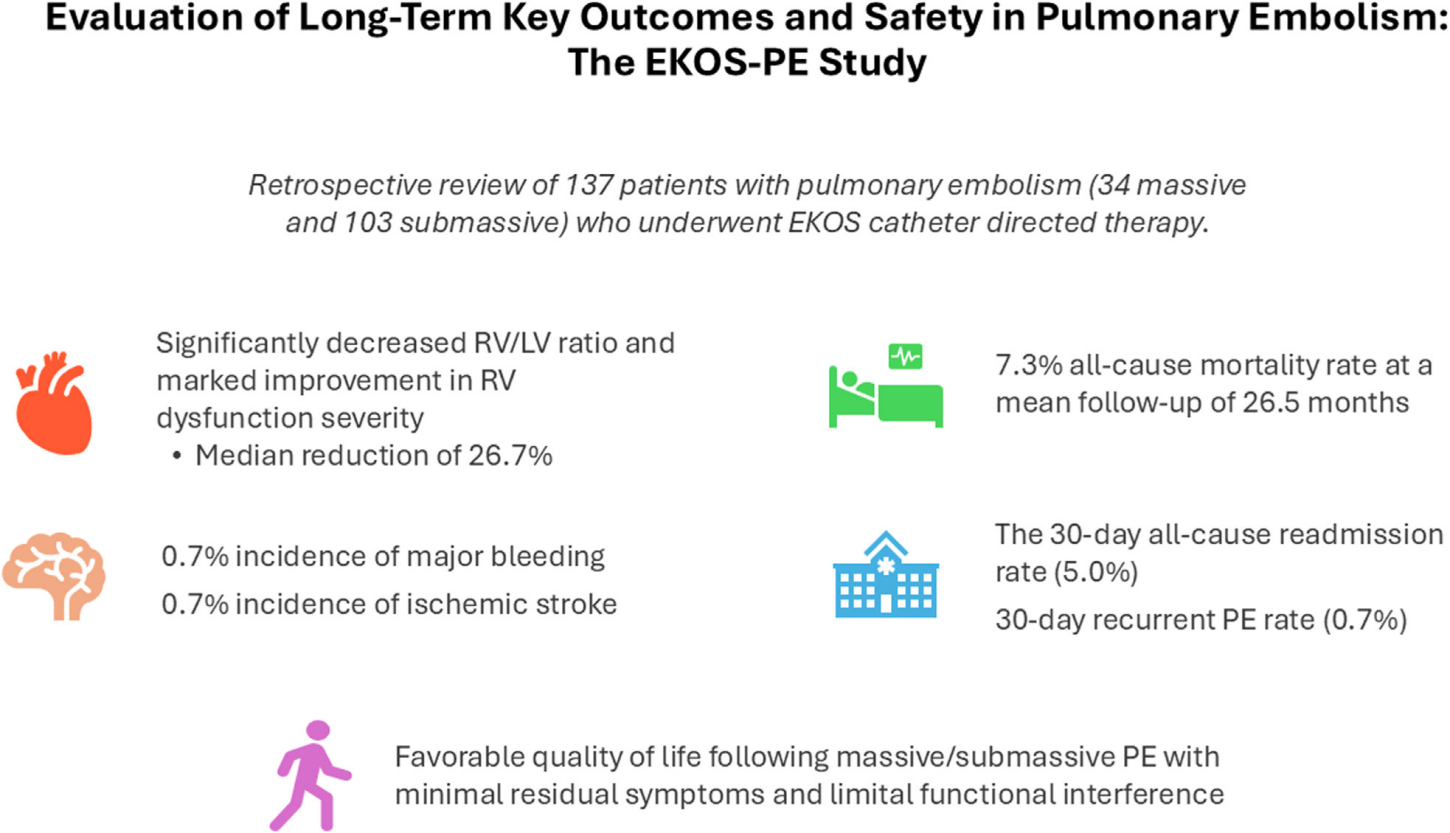
In patients with massive or submassive pulmonary embolism (PE) treated with ultrasound-assisted, catheter-directed thrombolysis, there was significant long-term improvement in right ventricular (RV) function, low mortality, and favorable patient-reported quality of life outcomes. These findings expand on current evidence supporting the EkoSonic Endovascular System (EKOS) as an effective and safe intervention for patients at high-risk or with contraindications to systemic thrombolysis. LV, left ventricular.

The significant reductions observed in RV strain, as indicated by the decreased RV/LV ratio as well as the notable improvement in the degree of RV dysfunction, underscore EKOS’s effectiveness in reversing the physiological impact of massive and submassive PE. Postintervention, the mean RV/LV ratio declined from 1.12 to 0.84, reflecting substantial long-term improvement in RV function (*P* < .01). These outcomes align with earlier findings from studies like SEATTLE II and OPTALYSE PE, which demonstrated short-term improvements in RV function following the EKOS treatment.^12^, ^13^, ^14^ Additionally, the long-term reduction in RV strain in our cohort suggests that the EKOS therapy may play an important role in reducing the incidence of chronic RV dysfunction and potentially mitigating other post-PE complications.

Quality of life was assessed using the PEmb-QoL questionnaire in a subset of patients. Although this was a one-time assessment due to the retrospective study design, the results are encouraging with 61.5% of participants rating their lung condition as “much better” now compared to before the procedure, and the majority reporting minimal or no limitations in daily activities (median score 3 [3-3]). Furthermore, 65.4% indicated no interference in their normal social activities, and 69.2% experienced no current pain around the shoulder blades or chest. It is important to note that without serial QoL assessments, we cannot draw definitive conclusions about improvements over time. Nevertheless, the reported scores suggest a low prevalence of persistent symptoms, indicating that the EKOS therapy may provide substantial relief from the lingering effects of PE.

Over an average follow-up period of 26.5 ± 17.2 months, the all-cause mortality rate in our cohort was 7.3%, with 1 death attributed to recurrent PE occurring more than 2 years postthrombolysis. Importantly, there were minimal major adverse events following the procedure with only 1 retroperitoneal bleeding episode and 1 stroke (without residual symptoms) during the index hospitalization. These findings emphasize the safety of the EKOS therapy, particularly for patients with contraindications to systemic fibrinolysis. The 30-day all-cause readmission rate was notably low at 5.1%, with only 1 readmission (0.7%) related to recurrent PE. These results highlight the potential of the EKOS therapy to achieve durable clinical stability in patients with massive and submassive PE, a population with mortality rates reaching up to 65%.^3^^,^^8^^,^^19^

Our findings mirror those of other contemporary trials such as the REAL-PE study, which reported a similar in-hospital mortality rate of approximately 3.7% for patients undergoing ultrasound-assisted CDT, with a 30-day readmission rate of 5.4%.^20^ Additionally, the PEERLESS randomized controlled trial, which compared CDT to large-bore mechanical thrombectomy in intermediate-risk PE patients, demonstrated similar mortality outcomes between the 2 approaches but identified fewer all-cause readmissions with large-bore mechanical thrombectomy (3.2% vs 7.9%).^21^ Despite the differences in methodology and patient populations, these findings collectively suggest that CDT, including EKOS, offers an effective intervention for reducing both immediate and long-term morbidity in PE patients while maintaining a favorable safety profile.

### Limitations

This study has several limitations that warrant consideration. As a retrospective analysis from a single health care system, our findings may be influenced by selection bias and may lack full generalizability to broader populations. Additionally, incomplete follow-up data for all patients could impact the precision of long-term outcome assessments. QoL data were available for only 38% (52/137) of patients, and the absence of serial evaluations further limits our ability to evaluate changes over time and to attribute improvements directly to the EKOS intervention. Variations in tissue plasminogen activator dosing and treatment duration may have additionally influenced the results, while the variable timing of postprocedure echocardiograms introduces another layer of potential variability. Future randomized controlled trials with multicenter enrollment and standardized, extended follow-up are necessary to validate these findings.

## Conclusion

The EkoSonic Endovascular System therapy for massive and submassive PE demonstrated significant, long-term improvements in RV function and a low all-cause mortality rate at a mean follow-up of 26.5 months. Among patients who completed QoL assessments, most reported minimal or no residual symptoms, little interference with daily activities, and perceived improvement in lung health. Ultimately, these findings support the effectiveness and safety of the EKOS therapy for high-risk PE, particularly in those with contraindications to systemic thrombolysis. Further prospective, multicenter studies are warranted to confirm these long-term benefits and optimize treatment strategies.

## References

[1] (2008). Office of the Surgeon General (US), National Heart, Lung, and Blood Institute (US). The Surgeon General’s Call to Action to Prevent Deep Vein Thrombosis and Pulmonary Embolism. Office of the Surgeon General (US).

[2] Russell C., Keshavamurthy S., Saha S. (2022). Classification and stratification of pulmonary embolisms. Int J Angiol.

[3] Bĕlohlávek J., Dytrych V., Linhart A. (2013). Pulmonary embolism, part I: epidemiology, risk factors and risk stratification, pathophysiology, clinical presentation, diagnosis and nonthrombotic pulmonary embolism. Exp Clin Cardiol.

[4] Klok F.A., van der Hulle T., den Exter PL den, Lankeit M., Huisman M.V., Konstantinides S. (2014). The post-PE syndrome: a new concept for chronic complications of pulmonary embolism. Blood Rev.

[5] Barritt D.W. (1964). The diagnosis and management of pulmonary embolism. Postgrad Med J.

[6] Minges K.E., Bikdeli B., Wang Y. (2015). National trends in pulmonary embolism hospitalization rates and outcomes for adults aged ≥65 years in the United States (1999 to 2010). Am J Cardiol.

[7] Giri J., Sista A.K., Weinberg I. (2019). Interventional therapies for acute pulmonary embolism: current status and principles for the development of novel evidence: a scientific statement from the American Heart Association. Circulation.

[8] Konstantinides S.V., Meyer G., Becattini C. (2020). 2019 ESC guidelines for the diagnosis and management of acute pulmonary embolism developed in collaboration with the European Respiratory Society (ERS). Eur Heart J.

[9] Meyer G., Vicaut E., Danays T. (2014). Fibrinolysis for patients with intermediate-risk pulmonary embolism. N Engl J Med.

[10] Jaff M.R., McMurtry M.S., Archer S.L. (2011). Management of massive and submassive pulmonary embolism, iliofemoral deep vein thrombosis, and chronic thromboembolic pulmonary hypertension: a scientific statement from the American Heart Association. Circulation.

[11] Mangi M.A., Rehman H., Bansal V., Zuberi O. (2017). Ultrasound assisted catheter-directed thrombolysis of acute pulmonary embolism: a review of current literature. Cureus.

[12] Kucher N., Boekstegers P., Müller O.J. (2014). Randomized, controlled trial of ultrasound-assisted catheter-directed thrombolysis for acute intermediate-risk pulmonary embolism. Circulation.

[13] Piazza G., Hohlfelder B., Jaff M.R. (2015). A prospective, single-arm, multicenter trial of ultrasound-facilitated, catheter-directed, low-dose fibrinolysis for acute massive and submassive pulmonary embolism: the SEATTLE II study. JACC Cardiovasc Interv.

[14] Tapson V.F., Sterling K., Jones N. (2018). A randomized trial of the optimum duration of acoustic pulse thrombolysis procedure in acute intermediate-risk pulmonary embolism: the OPTALYSE PE trial. JACC Cardiovasc Interv.

[15] EkoSonic system gains FDA clearance for pulmonary embolism treatment. Endovascular Today. https://www.evtoday.com/news/ekosonic-system-gains-fda-clearance-for-pulmonary-embolism-treatment.

[16] Robles K.E., Armbruster A.L., Austin S.E., Baker J.N. (2022). Utilization of EKOS in patients with pulmonary embolism. Innovations (Phila).

[17] Klok F.A., Cohn D.M., Middeldorp S. (2010). Quality of life after pulmonary embolism: validation of the PEmb-QoL Questionnaire. J Thromb Haemost.

[18] Yamashita Y., Morimoto T., Amano H. (2020). Validation of simplified PESI score for identification of low-risk patients with pulmonary embolism: from the COMMAND VTE Registry. Eur Heart J Acute Cardiovasc Care.

[19] Freund Y., Cohen-Aubart F., Bloom B. (2022). Acute pulmonary embolism: a review. JAMA.

[20] Monteleone P., Ahern R., Banerjee S. (2024). Modern treatment of pulmonary embolism (USCDT vs MT): results from a real-world, big data analysis (REAL-PE). J Soc Cardiovasc Angiogr Interv.

[21] Jaber W.A., Gonsalves C.F., Stortecky S. (2025). Large-bore mechanical thrombectomy versus catheter-directed thrombolysis in the management of intermediate-risk pulmonary embolism: primary results of the PEERLESS randomized controlled trial. Circulation.

