# Supplementary material for: Evaluation of Long-Term Key Outcomes and Safety in Pulmonary Embolism: The EKOS-PE Study
Source: J Soc Cardiovasc Angiogr Interv. 2025 Jul 23;4(8):103712. doi: 10.1016/j.jscai.2025.103712 (PMC12462068; doi:10.1016/j.jscai.2025.103712)
Supplement: Supplementary material [file mmc1.docx]

**Supplemental Material:**

**PEum-QOL Questionnaire Phone Script**

**Researcher:** Hello, may I please speak with Mr./Ms.**________,**

**If patient is not available.**

**Researcher**: My name is __________, I am calling from the Heart Hospital in Plano. Is there a good time to call back to speak with Mr./Ms. _______, or can I leave my call back information for Mr./Ms. _____? (If yes, Leave name and number and thank the person that answered the phone.)

**If available or on the phone.**

**Researcher:** My name is __________, I am calling from the Heart Hospital in Plano about a research study looking at the impact of a procedure done at THH that uses a catheter to dissolve blood clots and its long-term clinical outcomes. I wanted to know if you would be interested in taking part in this project. Taking part in this study is completely voluntary and will only take a few minutes of your time. (If the patient wonders why clinical outcomes are being examined: We are always wanting to improve what we know about what happens to our patients after they are discharged from the hospital. Recent studies showed the clinical outcomes after 6 months of intervention but long-term outcomes beyond this time frame remain largely unknown).

**Researcher:** Would you be interested in hearing more?

**If no:**

**Researcher:** Thank you for taking the time to hear me out today. I hope you have a great rest of your day.

**If yes:**

**Researcher:** If you choose to take part in this study, all of the information you may share with me will be kept confidential. There are no risks or benefits to you for being in this study and you have the option to not be in the study. I will be asking about 5-6 questions about things that may or may not have happened since your intervention. This will include information such as dates and locations for admission. Followed by this I will be asking you questions about your quality of life. Would you like to take part?

**If no:**

**Researcher:** Thank you for taking the time to hear me out today. I hope you have a great rest of your day.

**If yes:**

**Researcher:** Thank you for choosing to take part in this study. Can you confirm that your name is ____ and you had a procedure for your blood clots on _____ (date)? Have you have been hospitalized since your intervention? If so, where were you admitted and when? Have you had a stroke or TIA (mini stroke) since your surgery? If so, when did this happen? Have you been diagnosed with a pulmonary embolism since your surgery? If so, when did this happen? Have you been diagnosed with Pulmonary arterial hypertension since your surgery? If so, when did this happen?

Now, I’m going to ask you questions about your quality of life. There are 9 questions, and it will take about 5-10 minutes of your time. [Read attached PEmb-QoL questionnaire (attached) with 9 questions and record answers].

**After information is obtained**

**Researcher:** Thank you for your time today. Without the help of people like you, this important research would not be conducted.

**If Subject does not answer phone leave a voicemail:**

Hello Mr./Ms. ________,

My name is __________, I'm calling from The Heart Hospital in Plano about a research project we have underway. For more information, please contact us at XXX.XXX.XXXX."

**PEmb-QoL Questionnaire**

| 1. Since the procedure, how often have you had any of the following symptoms from your lungs? *(Circle 1 answer on each line)* | | | | | | | | | | | | |
| --- | --- | --- | --- | --- | --- | --- | --- | --- | --- | --- | --- | --- |
|  | **Every day** | **Several times a week** | | | **About once a week** | | **Less than once a week** | | | | | **Never** |
| Pain behind or between the shoulder blades? | 1 | 2 | | | 3 | | 4 | | | | | 5 |
| Pain on or in the chest? | 1 | 2 | | | 3 | | 4 | | | | | 5 |
| Pain in the back? | 1 | 2 | | | 3 | | 4 | | | | | 5 |
| Sensation of pressure? | 1 | 2 | | | 3 | | 4 | | | | | 5 |
| Feeling that there is ‘still something there’? | 1 | 2 | | | 3 | | 4 | | | | | 5 |
| ‘Burning sensation’ in the lungs? | 1 | 2 | | | 3 | | 4 | | | | | 5 |
| ‘Nagging feeling’ in the lungs? | 1 | 2 | | | 3 | | 4 | | | | | 5 |
| Difficulty in breathing or breathlessness? | 1 | 2 | | | 3 | | 4 | | | | | 5 |
| 2. At what time of day are your **lung symptoms most intense**? (circle *one answer*) | | | | | | | | | | | | |
| 1. On waking | | | | | | | | | | | | |
| 2. At mid-day | | | | | | | | | | | | |
| 3. At the end of the day | | | | | | | | | | | | |
| 4. During the night | | | | | | | | | | | | |
| 5. At any time of the day | | | | | | | | | | | | |
| 6. Never | | | | | | | | | | | | |
| 3. Compared to prior surgery, how would you rate the condition of your lungs in general now? (circle one answer) | | | | | | | | | | | | |
| 1. Much better now than before the surgery. | | | | | | | | | | | | |
| 2. Somewhat better now than 1 year ago | | | | | | | | | | | | |
| 3. About the same now as 1 year ago | | | | | | | | | | | | |
| 4. Somewhat worse now than 1 year ago | | | | | | | | | | | | |
| 5. Much worse now than 1 year ago | | | | | | | | | | | | |
| 6. I did not have any problems with my lungs | | | | | | | | | | | | |
| 4. The following items are about activities that you might do in a typical day. Do your lung symptoms now limit you in these activities? If so, how much? (Circle one answer on each line) | | | | | | | | | | | | |
|  | | **I do not work** | | | **YES, Limited A Lot** | | **YES, Limited A Little** | | | **NO, Not Limited At All** | | |
| a. **Daily activities at work** | | 0 | | | 1 | | 2 | | | 3 | | |
| b. **Daily activities at home** (e.g. housework, ironing,  doing odd jobs/repairs around the house, gardening, etc.…) | |  | | | 1 | | 2 | | | 3 | | |
| c. **Social or activities** (such as travelling, going to the cinema, parties, shopping) | |  | | | 1 | | 2 | | | 3 | | |
| d. **Vigorous activities**, such as running, lifting heavy objects, participating in strenuous sports | |  | | | 1 | | 2 | | | 3 | | |
| e. **Moderate activities**, such as moving a table, hoovering, swimming or cycling | |  | | | 1 | | 2 | | | 3 | | |
| f. Lifting or carrying groceries | |  | | | 1 | | 2 | | | 3 | | |
| g. Climbing **several** flights of stairs | |  | | | 1 | | 2 | | | 3 | | |
| h. Climbing **one** flight of stairs | |  | | | 1 | | 2 | | | 3 | | |
| i. Bending, kneeling, or squatting | |  | | | 1 | | 2 | | | 3 | | |
| j. Walking **more than half a mile** | |  | | | 1 | | 2 | | | 3 | | |
| k. Walking **a couple of hundred yards** | |  | | | 1 | | 2 | | | 3 | | |
| l. Walking **about one hundred yards** | |  | | | 1 | | 2 | | | 3 | | |
| m. Washing or dressing yourself | |  | | | 1 | | 2 | | | 3 | | |
| 5. Since the procedure, have you had any of the following problems with your work or other regular daily activities as a result of your **lung symptoms**? *(Circle one answer on each line)* | | | | | | | | | | | | |
|  | | | | | | | | | **YES** | | | **NO** |
| a. Cut down the **amount of time** you spent on work or other activities | | | | | | | | | 1 | | | 2 |
| b. **Accomplished less** than you would like | | | | | | | | | 1 | | | 2 |
| c. Were limited in the **kind** of work or other activities | | | | | | | | | 1 | | | 2 |
| d. Had **difficulty** performing the work or other activities (e.g. it took extra effort) | | | | | | | | | 1 | | | 2 |
| 6. At present, to what extent have your **lung symptoms** interfered with your normal social activities with family, friends, neighbors or groups? (Circle one answer) | | | | | | | | | | | | |
| 1. Not at all | | | | | | | | | 4. Quite a bit | | | |
| 2. Slightly | | | | | | | | | 5. Extremely | | | |
| 3. Moderately | | | | | | | | |  | | | |
| 7. How much **pain around your shoulder blades/pain in your chest** have you experienced during at present? (Circle one answer) | | | | | | | | | | | | |
| 1. None | | | 4. Quite a bit | | | | | | | | | |
| 2. Very slight | | | 5. Serious | | | | | | | | | |
| 3. Slight | | | 6. Very serious | | | | | | | | | |
| 8. How **much breathlessness** have you experienced at present? (Circle one answer) | | | | | | | | | | | | |
| 1. None | | | 4. Quite a bit | | | | | | | | | |
| 2. Very slight | | | 5. Serious | | | | | | | | | |
| 3. Slight | | | 6. Very serious | | | | | | | | | |
| 9. These questions are about how you feel and how things have been with you **during the past 4 weeks as a result of** **your lung symptoms**. For each question, please give the one answer that comes closest to the way you have been feeling. How much of the time during the **past 4 weeks** *(Circle one answer on each line)* | | | | | | | | | | | | |
|  | **All of the Time** | **Most of the Time** | | **A good Bit of the Time** | | **Some of the Time** | | **A Little of the Time** | | | **None of the Time** | |
| Were you worried about having another pulmonary embolism? | 1 | 2 | | 3 | | 4 | | 5 | | | 6 | |
| Did you feel irritable? | 1 | 2 | | 3 | | 4 | | 5 | | | 6 | |
| Would you have been worried if you had to stop taking anticoagulant medication? | 1 | 2 | | 3 | | 4 | | 5 | | | 6 | |
| Did you become emotional more readily? | 1 | 2 | | 3 | | 4 | | 5 | | | 6 | |
| Did it bother you that you became emotional more quickly? | 1 | 2 | | 3 | | 4 | | 5 | | | 6 | |
| Were you depressed or in low spirits? | 1 | 2 | | 3 | | 4 | | 5 | | | 6 | |
| Did you feel that you were a burden to your family and friends? | 1 | 2 | | 3 | | 4 | | 5 | | | 6 | |
| Were you afraid to exert yourself? | 1 | 2 | | 3 | | 4 | | 5 | | | 6 | |
| Did you feel limited in taking a trip? | 1 | 2 | | 3 | | 4 | | 5 | | | 6 | |
| Were you afraid of being alone? | 1 | 2 | | 3 | | 4 | | 5 | | | 6 | |

| **Supplemental Table 1.** Responses to PEum-QOL Questionnaire | |  |  |
| --- | --- | --- | --- |
| 1. *Since the procedure, how often have you had any of the following symptoms from your lungs? | N=52 | |  |
| Pain behind or between the shoulder blades? | 5 [5-5] | |  |
| Pain on or in the chest? | 5 [4-5] | |  |
| Pain in the back? | 5 [5-5] | |  |
| Sensation of pressure? | 5 [5-5] | |  |
| Feeling that there is ‘still something there’? | 5 [5-5] | |  |
| ‘Burning sensation’ in the lungs? | 5 [5-5] | |  |
| ‘Nagging feeling’ in the lungs? | 5 [5-5] | |  |
| Difficulty in breathing or breathlessness? | 5 [3-5] | |  |
| 2. At what time of day are your lung symptoms most intense? | N=52 | |  |
| 1. On waking | 7 (13.5) | |  |
| 2. At mid-day | 0 (0) | |  |
| 3. At the end of the day | 3 (5.8) | |  |
| 4. During the night | 2 (3.8) | |  |
| 5. At any time of the day | 12 (23.1) | |  |
| 6. Never | 26 (50) | |  |
| 3. Compared to prior surgery, how would you rate the condition of your lungs in general now? | N=52 | |  |
| 1. Much better now than before the surgery. | 32 (61.5) | |  |
| 2. Somewhat better now than 1 year ago | 4 (7.7) | |  |
| 3. About the same now as 1 year ago | 2 (5.9) | |  |
| 4. Somewhat worse now than 1 year ago | 1 (1.9) | |  |
| 5. Much worse now than 1 year ago | 0 (0) | |  |
| 6. I did not have any problems with my lungs | 7 (13.5) | |  |
| 4. †The following items are about activities that you might do in a typical day. Do your lung symptoms now limit you in these activities? If so, how much? | N=52 | |  |
| a. Daily activities at work | 3 [2-3] | |  |
| b. Daily activities at home (e.g. housework, ironing, doing odd jobs/repairs around the house, gardening, etc.…) | 3 [3-3] | |  |
| c. Social or activities (such as travelling, going to the cinema, parties, shopping) | 3 [3-3] | |  |
| d. Vigorous activities, such as running, lifting heavy objects, participating in strenuous sports | 3 [1-3] | |  |
| e. Moderate activities, such as moving a table, hoovering, swimming or cycling | 3 [2-3] | |  |
| f. Lifting or carrying groceries | 3 [2-3] | |  |
| g. Climbing several flights of stairs | 3 [1-3] | |  |
| h. Climbing one flight of stairs | 3 [1-3] | |  |
| i. Bending, kneeling, or squatting | 3 [3-3] | |  |
| j. Walking more than half a mile | 3 [2.5-3] | |  |
| k. Walking a couple of hundred yards | 3 [3-3] | |  |
| l. Walking about one hundred yards | 3 [3-3] | |  |
| m. Washing or dressing yourself | 3 [3-3] | |  |
| 5. Since the procedure, have you had any of the following problems with your work or other regular daily activities as a result of your lung symptoms? | N=52 | |  |
| a. Cut down the amount of time you spent on work or other activities | 10 (19.2) | |  |
| b. Accomplished less than you would like | 13 (25.0) | |  |
| c. Were limited in the kind of work or other activities | 14 (26.9) | |  |
| d. Had difficulty performing the work or other activities (e.g. it took extra effort) | 12 (23.1) | |  |
| 6. At present, to what extent have your lung symptoms interfered with your normal social activities with family, friends, neighbors or groups? | N=52 | |  |
| 1. Not at all | 34 (65.4) | |  |
| 2. Slightly | 5 (9.6) | |  |
| 3. Moderately | 8 (15.4) | |  |
| 4. Quite a bit | 1 (1.9) | |  |
| 5. Extremely | 0 (0) | |  |
| 7. How much pain around your shoulder blades/pain in your chest have you experienced during at present? | N=52 | |  |
| 1. None | 36 (69.2) | |  |
| 2. Very slight | 3 (5.8) | |  |
| 3. Slight | 7 (13.5) | |  |
| 4. Quite a bit | 3 (5.8) | |  |
| 5. Serious | 0 (0) | |  |
| 6. Very serious | 0 (0) | |  |
| 8. How much breathlessness have you experienced at present? | N=52 | |  |
| 1. None | 28 (53.8) | |  |
| 2. Very slight | 5 (9.6) | |  |
| 3. Slight | 10 (19.2) | |  |
| 4. Quite a bit | 5 (9.6) | |  |
| 5. Serious | 2 (3.8) | |  |
| 6. Very serious | 1 (1.9) | |  |
| 9. ‡These questions are about how you feel and how things have been with you during the past 4 weeks as a result of your lung symptoms. For each question, please give the one answer that comes closest to the way you have been feeling. How much of the time during the past 4 weeks: | N=52 | |  |
| Were you worried about having another pulmonary embolism? | 4 [3-6] | |  |
| Did you feel irritable? | 6 [5-6] | |  |
| Would you have been worried if you had to stop taking anticoagulant medication? | 4.5 [2-6] | |  |
| Did you become emotional more readily? | 6 [6-6] | |  |
| Did it bother you that you became emotional more quickly? | 6 [6-6] | |  |
| Were you depressed or in low spirits? | 6 [4.75-6] | |  |
| Did you feel that you were a burden to your family and friends? | 6 [6-6] | |  |
| Were you afraid to exert yourself? | 5 [2.75-6] | |  |
| Did you feel limited in taking a trip? | 6 [5-6] | |  |
| Were you afraid of being alone? | 6 [6-6] | |  |
| *Categorical data are described as n (%). Continuous data are presented as median [Interquartile Range (IQR)].*  ** 1=Everyday, 2=Several times a week, 3=About once a week, 4=Less than once a week, 5=Never*  *† 0=I do not work; 1=Yes, limited a lot; 2=Yes, limited a little; 3=No, not limited at all*  *‡ 1=All of the time, 2=Most of the time, 3=A good bit of the time, 4=Some of the time, 5=A little of the time, 6=None of the time* | | | |
